# Commentary: Evidence for human transmission of amyloid-β pathology and cerebral amyloid angiopathy

**DOI:** 10.3389/fnagi.2016.00005

**Published:** 2016-01-21

**Authors:** Isis Nem De Oliveira Souza, Evandro A. De-Souza

**Affiliations:** ^1^Institute of Biomedical Sciences, Federal University of Rio de JaneiroRio de Janeiro, Brazil; ^2^Department of Biophysics, Federal University of São PauloSão Paulo, Brazil

**Keywords:** Alzheimer's disease, Creutzfeldt-Jakob disease, prion, amyloid plaque, dementia, amyloidosis, cross-seeding, protein seed

With incubation time ranging from 5 to 40 years, Creutzfeldt-Jakob disease (CJD) is a deadly condition caused by the accumulation of toxic forms of the prion protein in the brain. Between the 50 s and 80 s, hundreds of patients developed CJD after being treated with cadaveric human growth hormone (c-hGH) contaminated with prion protein. This has led several countries to develop cohorts in order to follow these patients and understand the burden of prion disease in their lives over time.

Multiple studies were published linking amyloid-β (Aβ) deposits in the brain—a key feature of Alzheimer's disease (AD)—to prion disease patients that acquired it through several different sources (Watanabe and Duchen, 1993; Preusser et al., 2006; Tousseyn et al., 2015). In general, both Aβ deposits and prion protein deposits occurs by a nucleation-dependent polymerization process in which oligomeric “seed” proteins trigger the formation of an ordered nucleus that may rapidly grows into larger polymer fibrils (Harper and Lansbury, 1997). Recently, Jaunmuktane et al. (2015) performed an autopsy study providing evidence of a possible prion-like transmission of the amyloid-β protein in prion disease patients.

The work evaluated brain tissue from 8 patients that died between 36 and 51 years of age of iatrogenic CJD (iCJD) and found abnormal levels of Aβ protein in 7 of them. Such findings were intriguing since age-matched patients with different kinds of prion disease showed extremely rare occurrence of Aβ deposits, engaging researchers in a deeper investigation on the relationship between prion and Aβ proteins.

First, the authors excluded any genetic predisposition that these patients might have for several neurodegenerative diseases, including AD, amyotrophic lateral sclerosis, frontal temporal dementia and Parkinson's disease, screening for variations in ApoE and 16 other alleles. Such extensive screening wasn't performed in similar past studies (Hainfellner et al., 1998; Irwin et al., 2013). None of these patients presented causal mutations, but three of them presented possible risk factor alleles for frontotemporal dementia/AD (patient 4) and frontotemporal dementia (patients 6 and 8). Although Aβ deposits are not common in frontotemporal dementia, these patients were the ones presenting widespread cortical and leptomeningeal cerebral Aβ angiopathy. Anyhow, considering the large controversy relating genetic risk factors other than ApoE and actual AD development, such minor mutations seem negligible in the presented scenario.

Next, they compared the Aβ burden in these patients with other prion disease patients, in an attempt to connect the iatrogenically transmission specifically to Aβ burden. None of the CJD patients in the same age strata showed similar Aβ pathology using 4 different quantification methods, which the authors interpreted as an evidence of human transmission. However, the authors can't exclude the possibility of a stimulation of endogenous Aβ production by the prion protein, since other studies with larger cohorts found considerable amyloidopathy in the brain of sporadic CJD patients (Hainfellner et al., 1998; Irwin et al., 2013; Tousseyn et al., 2015). This argues against the idea of the Aβ burden being specific for iCJD.

Further, the authors checked for evidences of possible cross-seeding (when one abnormal protein—called a seed—is capable of modifying the folding of another type of protein) between the two protein deposits. Interestingly, cross-seeding with α-synuclein was already showed to occur with Aβ *in vitro* and with tau *in vivo* (Waxman and Giasson, 2011; Ono et al., 2012). The authors found an absence of co-localization between them both in plaques and in vascular deposits, suggesting that these pathologies might develop independently. However, as discussed, the lack of co-localization is not enough to completely exclude cross-seeding. Testing whether or not prion pathology might induce Aβ aggregation in health animals might be of interest. Also, they analyzed pituitary glands of AD patients for Aβ and further supported the possibility of contamination of c-GH batches with Aβ as well as prion proteins, as previously described (Peden et al., 2007). The authors argued that both proteins might have been present in the final preparation and iatrogenically contaminated the patients. A biochemical analysis of residual batches of c-GH for Aβ as well as their use in animal models of propagation could shed light into the matter.

Although Aβ is a hallmark of Alzheimer's disease, no tau pathology was found in the brain of such patients. The authors suggested that, had the patients not died of prion disease, the tau accumulation might have developed over time. It is indeed known that tau pathology develops after the Aβ deposits in AD (Jack et al., 2010), but with the presented findings is still uncertain if they would or not develop tau pathology and AD.

Their hypothesis is supported by the fact that iatrogenic transmission of Aβ has been demonstrated more than once in mice by both intracerebral and peripheral routes (Meyer-Luehmann et al., 2006; Eisele et al., 2009, 2010; Stöhr et al., 2012). Also, Tousseyn et al. (2015) exposed human brain homogenates to sporadic CJD human brain homogenates and found positive Aβ labeling as well as hyperphosphorilated tau accumulation. Recently, Ye et al. (2015) demonstrated that such seeds may stay dormant in a mouse brain for months and still be pathological once inoculated in another animal. It would be interesting to see a parabiosis experiment showing that cerebral Aβ deposits in transgenic mice, for example, could be transmitted to another, health, mice through the blood.

Collectively, the literature still doesn't support prion-like transmission as a relevant form of developing AD. Since other larger studies found controversial results, the next step to test Jaunmuktane et al. (2015) hypothesis would be to obtain data from larger cohorts of CJD patients. Once confirmed, we can imagine two scenarios: a scientific fun-fact with little epidemiological significance for the general population or a revolution in AD prevention, patient management and treatment development. Let's wait for the next chapter.

## Author contributions

IS wrote the initial draft. ED revised the initial draft and contributed with further writing. Both collected data from literature and revised the final manuscript.

### Conflict of interest statement

The authors declare that the research was conducted in the absence of any commercial or financial relationships that could be construed as a potential conflict of interest.
